# Independent and combined effects of improved water, sanitation, and hygiene, and improved complementary feeding, on child stunting and anaemia in rural Zimbabwe: a cluster-randomised trial

**DOI:** 10.1016/S2214-109X(18)30374-7

**Published:** 2018-12-13

**Authors:** Jean H Humphrey, Mduduzi N N Mbuya, Robert Ntozini, Lawrence H Moulton, Rebecca J Stoltzfus, Naume V Tavengwa, Kuda Mutasa, Florence Majo, Batsirai Mutasa, Goldberg Mangwadu, Cynthia M Chasokela, Ancikaria Chigumira, Bernard Chasekwa, Laura E Smith, James M Tielsch, Andrew D Jones, Amee R Manges, John A Maluccio, Andrew J Prendergast, Sanitation Hygiene Infant Nutrition Efficacy (SHINE) Trial Team, Jean H Humphrey, Andrew D Jones, Amee Manges, Goldberg Mangwadu, John A Maluccio, Mduduzi N N Mbuya, Lawrence H Moulton, Robert Ntozini, Andrew J Prendergast, Rebecca J Stoltzfus, James M Tielsch, Cynthia Chasokela, Ancikaria Chigumira, William Heylar, Preston Hwena, George Kembo, Florence D Majo, Batsirai Mutasa, Kuda Mutasa, Philippa Rambanepasi, Virginia Sauramba, Naume V Tavengwa, Franne Van Der Keilen, Chipo Zambezi, Dzivaidzo Chidhanguro, Dorcas Chigodora, Joseph F Chipanga, Grace Gerema, Tawanda Magara, Mandava Mandava, Tafadzwa Mavhudzi, Clever Mazhanga, Grace Muzaradope, Marian T Mwapaura, Simon Phiri, Alice Tengende, Cynthia Banda, Bernard Chasekwa, Leah Chidamba, Theodore Chidawanyika, Elisha Chikwindi, Lovemore K Chingaona, Courage K Chiorera, Adlight Dandadzi, Margaret Govha, Hlanai Gumbo, Karen T Gwanzura, Sarudzai Kasaru, Rachel Makasi, Alois M Matsika, Diana Maunze, Exevia Mazarura, Eddington Mpofu, Johnson Mushonga, Tafadzwa E Mushore, Tracey Muzira, Netsai Nembaware, Sibongile Nkiwane, Penias Nyamwino, Sandra D Rukobo, Thompson Runodamoto, Shepherd Seremwe, Pururudzai Simango, Joice Tome, Blessing Tsenesa, Umali Amadu, Beauty Bangira, Daniel Chiveza, Priscilla Hove, Horaiti A Jombe, Didymus Kujenga, Lenin Madhuyu, Prince M Makoni, Naume Maramba, Betty Maregere, Ellen Marumani, Elisha Masakadze, Phathisiwe Mazula, Caroline Munyanyi, Grace Musanhu, Raymond C Mushanawani, Sibongile Mutsando, Felicia Nazare, Moses Nyarambi, Wellington Nzuda, Trylife Sigauke, Monica Solomon, Tendai Tavengwa, Farisai Biri, Misheck Chafanza, Cloud Chaitezvi, Tsundukani Chauke, Collen Chidzomba, Tawanda Dadirai, Clemence Fundira, Athanasios C Gambiza, Tatenda Godzongere, Maria Kuona, Tariro Mafuratidze, Idah Mapurisa, Tsitsi Mashedze, Nokuthula Moyo, Charles Musariri, Matambudzo Mushambadope, Tawanda R Mutsonziwa, Augustine Muzondo, Rudo Mwareka, Juleika Nyamupfukudza, Baven Saidi, Tambudzai Sakuhwehwe, Gerald Sikalima, Jenneth Tembe, Tapiwanashe E Chekera, Owen Chihombe, Muchaneta Chikombingo, Tichaona Chirinda, Admire Chivizhe, Ratidzai Hove, Rudo Kufa, Tatenda F Machikopa, Wilbert Mandaza, Liberty Mandongwe, Farirai Manhiyo, Emmanuel Manyaga, Peter Mapuranga, Farai S Matimba, Patience Matonhodze, Sarah Mhuri, Joice Mike, Bekezela Ncube, Walter T S Nderecha, Munyaradzi Noah, Charles Nyamadzawo, Jonathan Penda, Asinje Saidi, Sarudzai Shonhayi, Clemence Simon, Monica Tichagwa, Rachael Chamakono, Annie Chauke, Andrew F Gatsi, Blessing Hwena, Hillary Jawi, Benjamin Kaisa, Sithembile Kamutanho, Tapiwa Kaswa, Paradhi Kayeruza, Juliet Lunga, Nomatter Magogo, Daniel Manyeruke, Patricia Mazani, Fungai Mhuriyengwe, Farisai Mlambo, Stephen Moyo, Tawanda Mpofu, Mishelle Mugava, Yvonne Mukungwa, Fungai Muroyiwa, Eddington Mushonga, Selestino Nyekete, Tendai Rinashe, Kundai Sibanda, Milton Chemhuru, Jeffrey Chikunya, Vimbai F Chikwavaire, Charity Chikwiriro, Anderson Chimusoro, Jotam Chinyama, Gerald Gwinji, Nokuthula Hoko-Sibanda, Rutendo Kandawasvika, Tendai Madzimure, Brian Maponga, Antonella Mapuranga, Joana Marembo, Luckmore Matsunge, Simbarashe Maunga, Mary Muchekeza, Monica Muti, Marvin Nyamana, Efa Azhuda, Urayai Bhoroma, Ailleen Biriyadi, Elizabeth Chafota, Angelline Chakwizira, Agness Chamhamiwa, Tavengwa Champion, Stella Chazuza, Beauty Chikwira, Chengeto Chingozho, Abigail Chitabwa, Annamary Dhurumba, Albert Furidzirai, Andrew Gandanga, Chipo Gukuta, Beauty Macheche, Bongani Marihwi, Barbara Masike, Eunice Mutangandura, Beatrice Mutodza, Angeline Mutsindikwa, Alice Mwale, Rebecca Ndhlovu, Norah Nduna, Cathrine Nyamandi, Elias Ruvata, Babra Sithole, Rofina Urayai, Bigboy Vengesa, Micheal Zorounye, Memory Bamule, Michael Bande, Kumbirai Chahuruva, Lilian Chidumba, Zvisinei Chigove, Kefas Chiguri, Susan Chikuni, Ruvarashe Chikwanda, Tarisai Chimbi, Micheal Chingozho, Olinia Chinhamo, Regina Chinokuramba, Chiratidzo Chinyoka, Xaviour Chipenzi, Raviro Chipute, Godfrey Chiribhani, Mary Chitsinga, Charles Chiwanga, Anamaria Chiza, Faith Chombe, Memory Denhere, Ephania Dhamba, Miriam Dhamba, Joyas Dube, Florence Dzimbanhete, Godfrey Dzingai, Sikhutele Fusira, Major Gonese, Johnson Gota, Kresencia Gumure, Phinias Gwaidza, Margret Gwangwava, Winnet Gwara, Melania Gwauya, Maidei Gwiba, Joyce Hamauswa, Sarah Hlasera, Eustina Hlukani, Joseph Hotera, Lovemore Jakwa, Gilbert Jangara, Micheal Janyure, Christopher Jari, Duvai Juru, Tabeth Kapuma, Paschalina Konzai, Moly Mabhodha, Susan Maburutse, Chipo Macheka, Tawanda Machigaya, Florence Machingauta, Eucaria Machokoto, Evelyn Madhumba, Learnard Madziise, Clipps Madziva, Mavis Madzivire, Mistake Mafukise, Marceline Maganga, Senzeni Maganga, Emmanuel Mageja, Miriam Mahanya, Evelyn Mahaso, Sanelisiwe Mahleka, Pauline Makanhiwa, Mavis Makarudze, Constant Makeche, Nickson Makopa, Ranganai Makumbe, Mascline Mandire, Eunice Mandiyanike, Eunice Mangena, Farai Mangiro, Alice Mangwadu, Tambudzai Mangwengwe, Juliet Manhidza, Farai Manhovo, Irene Manono, Shylet Mapako, Evangelista Mapfumo, Timothy Mapfumo, Jane Mapuka, Douglas Masama, Getrude Masenge, Margreth Mashasha, Veronica Mashivire, Moses Matunhu, Pazvichaenda Mavhoro, Godfrey Mawuka, Ireen Mazango, Netsai Mazhata, David Mazuva, Mary Mazuva, Filomina Mbinda, John Mborera, Upenyu Mfiri, Florence Mhandu, Chrispen Mhike, Tambudzai Mhike, Artwell Mhuka, Judith Midzi, Siqondeni Moyo, Michael Mpundu, Nicholas Msekiwa, Dominic Msindo, Choice Mtisi, Gladys Muchemwa, Nyadziso Mujere, Ellison Mukaro, Kilvera Muketiwa, Silvia Mungoi, Esline Munzava, Rosewita Muoki, Harugumi Mupura, Evelyn Murerwa, Clarieta Murisi, Letwin Muroyiwa, Musara Muruvi, Nelson Musemwa, Christina Mushure, Judith Mutero, Philipa Mutero, Patrick Mutumbu, Cleopatra Mutya, Lucia Muzanango, Martin Muzembi, Dorcus Muzungunye, Valeliah Mwazha, Thembeni Ncube, Takunda Ndava, Nomvuyo Ndlovu, Pauline Nehowa, Dorothy Ngara, Leonard Nguruve, Petronella Nhigo, Samukeliso Nkiwane, Luckson Nyanyai, Judith Nzombe, Evelyn Office, Beatrice Paul, Shambadzirai Pavari, Sylvia Ranganai, Stella Ratisai, Martha Rugara, Peter Rusere, Joyce Sakala, Prosper Sango, Sibancengani Shava, Margaret Shekede, Cornellious Shizha, Tedla Sibanda, Neria Tapambwa, John Tembo, Netsai Tinago, Violet Tinago, Theresa Toindepi, John Tovigepi, Modesta Tuhwe, Kundai Tumbo, Tinashe Zaranyika, Tongai Zaru, Kamurayi Zimidzi, Matilda Zindo, Maria Zindonda, Nyaradzai Zinhumwe, Loveness Zishiri, Emerly Ziyambi, James Zvinowanda, Ekenia Bepete, Christine Chiwira, Naume Chuma, Abiegirl Fari, Samson Gavi, Violet Gunha, Fadzai Hakunandava, Constance Huku, Given Hungwe, Grace Maduke, Elliot Manyewe, Tecla Mapfumo, Innocent Marufu, Chenesai Mashiri, Shellie Mazenge, Euphrasia Mbinda, Abigail Mhuri, Charity Muguti, Lucy Munemo, Loveness Musindo, Laina Ngada, Dambudzo Nyembe, Rachel Taruvinga, Emma Tobaiwa, Selina Banda, Jesca Chaipa, Patricia Chakaza, Macdonald Chandigere, Annie Changunduma, Chenesai Chibi, Otilia Chidyagwai, Elika Chidza, Nora Chigatse, Lennard Chikoto, Vongai Chingware, Jaison Chinhamo, Marko Chinhoro, Answer Chiripamberi, Esther Chitavati, Rita Chitiga, Nancy Chivanga, Tracy Chivese, Flora Chizema, Sinikiwe Dera, Annacolleta Dhliwayo, Pauline Dhononga, Ennia Dimingo, Memory Dziyani, Tecla Fambi, Lylian Gambagamba, Sikangela Gandiyari, Charity Gomo, Sarah Gore, Jullin Gundani, Rosemary Gundani, Lazarus Gwarima, Cathrine Gwaringa, Samuel Gwenya, Rebecca Hamilton, Agnes Hlabano, Ennie Hofisi, Florence Hofisi, Stanley Hungwe, Sharai Hwacha, Aquiiline Hwara, Ruth Jogwe, Atanus Kanikani, Lydia Kuchicha, Mitshel Kutsira, Kumbulani Kuziyamisa, Mercy Kuziyamisa, Benjamin Kwangware, Portia Lozani, Joseph Mabuto, Vimbai Mabuto, Loveness Mabvurwa, Rebecca Machacha, Cresenzia Machaya, Roswitha Madembo, Susan Madya, Sheneterai Madzingira, Lloyd Mafa, Fungai Mafuta, Jane Mafuta, Alfred Mahara, Sarudzai Mahonye, Admire Maisva, Admire Makara, Margreth Makover, Ennie Mambongo, Murenga Mambure, Edith Mandizvidza, Gladys Mangena, Elliot Manjengwa, Julius Manomano, Maria Mapfumo, Alice Mapfurire, Letwin Maphosa, Jester Mapundo, Dorcas Mare, Farai Marecha, Selina Marecha, Christine Mashiri, Medina Masiya, Thembinkosi Masuku, Priviledge Masvimbo, Saliwe Matambo, Getrude Matarise, Loveness Matinanga, John Matizanadzo, Margret Maunganidze, Belinda Mawere, Chipiwa Mawire, Yulliana Mazvanya, Maudy Mbasera, Magret Mbono, Cynthia Mhakayakora, Nompumelelo Mhlanga, Bester Mhosva, Nomuhle Moyo, Over Moyo, Robert Moyo, Charity Mpakami, Rudo Mpedzisi, Elizabeth Mpofu, Estery Mpofu, Mavis Mtetwa, Juliet Muchakachi, Tsitsi Mudadada, Kudakwashe Mudzingwa, Mejury Mugwira, Tarsisio Mukarati, Anna Munana, Juliet Munazo, Otilia Munyeki, Patience Mupfeka, Gashirai Murangandi, Maria Muranganwa, Josphine Murenjekwa, Nothando Muringo, Tichafara Mushaninga, Florence Mutaja, Dorah Mutanha, Peregia Mutemeri, Beauty Mutero, Edina Muteya, Sophia Muvembi, Tandiwe Muzenda, Agnes Mwenjota, Sithembisiwe Ncube, Tendai Ndabambi, Nomsa Ndava, Elija Ndlovu, Eveln Nene, Enniah Ngazimbi, Atalia Ngwalati, Tafirenyika Nyama, Agnes Nzembe, Eunica Pabwaungana, Sekai Phiri, Ruwiza Pukuta, Melody Rambanapasi, Tambudzai Rera, Violet Samanga, Sinanzeni Shirichena, Chipiwa Shoko, More Shonhe, Cathrine Shuro, Juliah Sibanda, Edna Sibangani, Nikisi Sibangani, Norman Sibindi, Mercy Sitotombe, Pearson Siwawa, Magret Tagwirei, Pretty Taruvinga, Antony Tavagwisa, Esther Tete, Yeukai Tete, Elliot Thandiwe, Amonilla Tibugari, Stella Timothy, Rumbidzai Tongogara, Lancy Tshuma, Mirirayi Tsikira, Constance Tumba, Rumbidzayi Watinaye, Ethel Zhiradzango, Esther Zimunya, Leanmary Zinengwa, Magret Ziupfu, Job Ziyambe, James A Church, Amy Desai, Dadirai Fundira, Ethan Gough, Rukundo A Kambarami, Cynthia R Matare, Thokozile R Malaba, Tatenda Mupfudze, Francis Ngure, Laura E Smith, Val Curtis, Katherine L Dickin, Jean-Pierre Habicht, Collen Masimirembwa, Peter Morgan, Gretel H Pelto, Corinne Sheffner-Rogers, Roslyn Thelingwani, Paul Turner, Lindiwe Zungu, Tariro Makadzange, Hilda A Mujuru, Chandiwana Nyachowe, Rugare Chakadai, Gabriel Chanyau, Mary G Makamure, Humphrey Chiwariro, Tambudzai Mtetwa, Jeffrey Chikunya, Lisbern Maguwu, Simon Nyadundu, Tshebukani Moyo, Beauty Chayima, Lucy Mvindi, Pauline Rwenhamo, Shamiso Muzvarwandoga, Rumbidzai Chimukangara, Handrea Njovo, Talent Makoni

**Affiliations:** a Department of International Health, Johns Hopkins Bloomberg School of Public Health, Baltimore MD, USA; b Zvitambo Institute for Maternal and Child Health Research, Harare, Zimbabwe; c Division of Nutritional Sciences, Cornell University, Ithaca, NY, USA; d Global Alliance for Improved Nutrition, Washington, DC, USA; e Ministry of Health and Child Care, Harare, Zimbabwe; f Department of Epidemiology and Environmental Health, School of Public Health and Health Professions, University at Buffalo, Buffalo, NY, USA; g Department of Global Health, Milken Institute School of Public Health, George Washington University, Washington, DC, USA; h Department of Nutritional Sciences, School of Public Health, University of Michigan, Ann Arbor, MI, USA; i University of British Columbia, Vancouver, BC, Canada; j Middlebury College, Middlebury, VT, USA; k Blizard Institute, Queen Mary University of London, London, UK

## Abstract

**Background:**

Child stunting reduces survival and impairs neurodevelopment. We tested the independent and combined effects of improved water, sanitation, and hygiene (WASH), and improved infant and young child feeding (IYCF) on stunting and anaemia in in Zimbabwe.

**Methods:**

We did a cluster-randomised, community-based, 2 × 2 factorial trial in two rural districts in Zimbabwe. Clusters were defined as the catchment area of between one and four village health workers employed by the Zimbabwe Ministry of Health and Child Care. Women were eligible for inclusion if they permanently lived in clusters and were confirmed pregnant. Clusters were randomly assigned (1:1:1:1) to standard of care (52 clusters), IYCF (20 g of a small-quantity lipid-based nutrient supplement per day from age 6 to 18 months plus complementary feeding counselling; 53 clusters), WASH (construction of a ventilated improved pit latrine, provision of two handwashing stations, liquid soap, chlorine, and play space plus hygiene counselling; 53 clusters), or IYCF plus WASH (53 clusters). A constrained randomisation technique was used to achieve balance across the groups for 14 variables related to geography, demography, water access, and community-level sanitation coverage. Masking of participants and fieldworkers was not possible. The primary outcomes were infant length-for-age Z score and haemoglobin concentrations at 18 months of age among children born to mothers who were HIV negative during pregnancy. These outcomes were analysed in the intention-to-treat population. We estimated the effects of the interventions by comparing the two IYCF groups with the two non-IYCF groups and the two WASH groups with the two non-WASH groups, except for outcomes that had an important statistical interaction between the interventions. This trial is registered with ClinicalTrials.gov, number NCT01824940.

**Findings:**

Between Nov 22, 2012, and March 27, 2015, 5280 pregnant women were enrolled from 211 clusters. 3686 children born to HIV-negative mothers were assessed at age 18 months (884 in the standard of care group from 52 clusters, 893 in the IYCF group from 53 clusters, 918 in the WASH group from 53 clusters, and 991 in the IYCF plus WASH group from 51 clusters). In the IYCF intervention groups, the mean length-for-age Z score was 0·16 (95% CI 0·08–0·23) higher and the mean haemoglobin concentration was 2·03 g/L (1·28–2·79) higher than those in the non-IYCF intervention groups. The IYCF intervention reduced the number of stunted children from 620 (35%) of 1792 to 514 (27%) of 1879, and the number of children with anaemia from 245 (13·9%) of 1759 to 193 (10·5%) of 1845. The WASH intervention had no effect on either primary outcome. Neither intervention reduced the prevalence of diarrhoea at 12 or 18 months. No trial-related serious adverse events, and only three trial-related adverse events, were reported.

**Interpretation:**

Household-level elementary WASH interventions implemented in rural areas in low-income countries are unlikely to reduce stunting or anaemia and might not reduce diarrhoea. Implementation of these WASH interventions in combination with IYCF interventions is unlikely to reduce stunting or anaemia more than implementation of IYCF alone.

**Funding:**

Bill & Melinda Gates Foundation, UK Department for International Development, Wellcome Trust, Swiss Development Cooperation, UNICEF, and US National Institutes of Health.

## Introduction

Globally, linear growth faltering (ie, stunting) is the most prevalent form of undernutrition.^1^, ^2^ Stunting largely occurs between conception and age 24 months, when mean length-for-age Z scores among Asian and African children plummet to −2·0, with little change thereafter.^1^, ^2^ Stunting reduces child survival, educational attainment, and adult economic productivity.^1^, ^2^ Furthermore, the offspring of adults who were stunted as children are at increased risk of stunting, creating an intergenerational cycle of low human capital.^1^, ^2^ Stunting has been largely intractable to targeted interventions. On average, complementary feeding interventions increase length-for-age Z scores by 0·1,^3^ and elimination of all diarrhoea in the first 2 years of life is estimated to increase length by 0·38 cm (or length-for-age Z scores by 0·13).^4^ Similar to stunting, childhood anaemia is prevalent among children younger than 2 years in Africa and Asia, and is a primary cause of cognitive delay.^2^ Increasing dietary iron intake reduces anaemia by 32–62%, which leaves a substantial proportion of disease unaddressed.^2^

**Research in context**

**Evidence before this study**

Before this trial, a Review done for *The Lancet* Nutrition Series highlighted that child stunting is a highly prevalent condition with adverse short-term and long-term sequelae. A systematic review of the literature showed that complementary feeding studies of improvements to the quality or quantity of infant diet could improve linear growth, but only moderately. That review was followed by an updated systematic review published just after our trial was completed; it showed that complementary feeding had an effect of 0·11 on length-for-age Z scores in food-secure populations, which is about 5–10% of the deficit experienced by Asian and African children. Finally, in seminal research done in The Gambia, child linear growth failure was strongly associated with indicators of environmental enteric dysfunction—increased gut permeability and systemic inflammation resulting from translocation of gut microbes. Although untested, the cause of environmental enteric dysfunction has been widely attributed to faecal–oral exposure resulting from living in conditions of poor water, sanitation, and hygiene (WASH). Before this trial, a meta-analysis of data from Demographic and Health Surveys for low-income and middle-income countries showed an association between linear growth and sanitation, but no randomised trials had been published in which the effect of sanitation on any child health outcome, including diarrhoea, had been tested. Additionally, many trials of handwashing with soap and chlorination of drinking water showed reductions in diarrhoea, but none reported the effects of these interventions on gut health or child growth. Since we began this trial, four published trials have assessed the effect of community-based sanitation on stunting. In two of these trials, both done in India, uptake of sanitation was low, the frequency of open defecation remained high, and no benefits for child health were reported in either. In a trial done in Indonesia, intervention uptake was modest. The intervention reduced diarrhoea but had no effect on linear growth. Finally, in a trial done in Mali, the intervention nearly doubled latrine coverage and substantially reduced the frequency of open defecation. It had no effect on diarrhoea, but increased length-for-age Z scores by 0·18. Most recently, the WASH Benefits trials in Bangladesh and Kenya tested the effect of six interventions (water treatment, handwashing, sanitation, all three WASH interventions together, infant feeding, and infant feeding plus all three WASH interventions) on linear growth and diarrhoea. In both trials, modest reductions in stunting were noted in the infant feeding group and the infant feeding plus WASH group, but no effects on linear growth were noted in any of the WASH-only group. Diarrhoea was reduced in all active groups in the Bangladesh trial except in the water treatment only group. None of the interventions reduced diarrhoea in the Kenya trial.

**Added value of this study**

The Sanitation Hygiene Infant Nutrition Efficacy (SHINE) trial was a 2 × 2 factorial cluster-randomised trial to test the independent and combined effects of improving infant diet and household WASH on length-for-age Z score and haemoglobin (primary outcomes) at 18 months of age. The WASH intervention included a play space to minimise geophagia and ingestion of chicken faeces by children in addition to conventional WASH interventions (sanitation, water treatment, handwashing, and hygienic preparation of food). The infant and young child feeding intervention included 20 g of a small-quantity lipid-based nutrient supplement per day from 6 to 18 months in addition to counselling that targeted key barriers to optimal infant feeding in this context. Intervention intensity (ie, frequency of contact between behaviour-change promoters and participants) was monthly. The IYCF intervention increased the mean length-for-age Z score by 0·16 (95% CI 0·08–0·23) and the mean haemoglobin concentration by 2·0 g/L (1·3–2·8), reduced stunting and anaemia, and improved ponderal growth compared with the non-IYCF interventions. The WASH intervention had no effect on these outcomes. Neither intervention reduced child diarrhoea or mortality.

**Implications of all the available evidence**

Our trial provides further high-level evidence that elementary WASH interventions (ie, provision of point-of-use water chlorination, handwashing stations not connected to water supply, and improved pit latrines, with promotion of hygiene behaviours) delivered at the household level in rural areas of low-income countries are unlikely to reduce stunting and might not reduce diarrhoea. Implementation of these WASH interventions together with IYCF interventions will not reduce stunting more than implementation of IYCF alone.

The UNICEF framework for undernutrition has been a guiding document for nearly 30 years.^5^ It highlights inadequate dietary intake and disease as the immediate causes of child undernutrition, and specifies that a multisectoral approach that addresses both proximal and distal determinants is required. Based on this framework, integration of improved infant diets with improved water, sanitation, and hygiene (WASH) is a logical approach, because of the role of WASH in reducing morbidity, especially diarrhoea. Along with others,^6^ we^7^ further hypothesised that the adverse effects of poor WASH on growth are primarily mediated through environmental enteric dysfunction, a subclinical condition of the small intestine characterised by blunted villi, intestinal inflammation, and intestinal permeability.^7^ Environmental enteric dysfunction reduces nutrient absorption, triggers chronic systemic inflammation, and is seemingly ubiquitous among people living in impoverished conditions in low-income and middle-income countries. We further hypothesised that a household WASH intervention targeting pathways of faecal–oral exposure in young children will reduce environmental enteric dysfunction, increase linear growth, and reduce anaemia by facilitating iron mobilisation and erythropoiesis. Finally, we hypothesised that the beneficial effects of WASH on growth and anaemia will be additive to those of improving infant diets.^2^ The Sanitation, Hygiene, Infant Nutrition Efficacy (SHINE) trial was designed to test these hypotheses.

## Methods

### Study design and participants

The design and methods of SHINE have been reported previously,^2^ and the protocol and statistical analysis plan are available online. Briefly, SHINE was a cluster-randomised, community-based 2 × 2 factorial trial in two contiguous rural districts in Zimbabwe (Chirumanzu and Shurugwi). The districts have a 15% prevalence of antenatal HIV^8^ and a high prevalence of schistosomiasis, but a very low prevalence of soil-transmitted helminths.^9^ Rotavirus vaccination was introduced during the trial from May, 2014. Households are usually single-family dwellings surrounded by farm land. Before the trial, mean distance between households was 82·6 m,^10^ and population density was 18·6 people per km^2^. Clusters were defined as the catchment area of between one and four village health workers (VHWs) employed by the Zimbabwe Ministry of Health and Child Care. Urban and uninhabited areas were excluded. Between Nov 22, 2012 and March 27, 2015, VHWs prospectively surveyed new pregnancies, established the date of last menstrual period, and referred pregnant women to SHINE research nurses, who enrolled eligible women. Women were eligible for inclusion if they permanently resided in a study cluster and were confirmed pregnant. During the recruitment period, the cutoff of gestational age for eligibility was gradually loosened (from 14 weeks' gestational age to just before parturition) to maximise recruitment (appendix). The Medical Research Council of Zimbabwe and the Institutional Review Board of the Johns Hopkins Bloomberg School of Public Health approved the study protocol. All participants provided written informed consent.

### Randomisation and masking

Clusters were allocated (1:1:1:1) to one of four treatment groups: standard of care, infant and young child feeding (IYCF), WASH, or IYCF plus WASH. LHM, the study's senior statistician, used a constrained randomisation technique^11^ to identify 5000 allocation schemes that achieved balance across the groups for 14 variables related to geography, demography, water access, and sanitation coverage, and also met bias and validity specifications (appendix). From these, ten allocations were randomly selected. The final allocation was selected at a public randomisation event attended by elected representatives of the study districts. Masking of participants and fieldworkers was not possible because of the obvious visual differences between interventions, but investigators were blinded to treatment groups until the final analysis of each prespecified outcome.

### Procedures

Interventions were informed by extensive formative research and piloting.^12^, ^13^, ^14^, ^15^ Behaviour-change modules were delivered by group-specific VHWs, who underwent training for 20 days to deliver standard of care, 30 days to deliver WASH, 32 days to deliver IYCF, and 35 days to deliver IYCF plus WASH. All enrolled women were scheduled to receive 15 behaviour-change modules with specific messages and interactive tools from enrolment until 12 months after the birth of their children (roughly one visit per month). Other family members were also encouraged to participate. A sequential integrated longitudinal intervention was delivered, and at each visit, previous information was reviewed before new information was introduced. Previously missed modules were delivered before any new material. Between 13 months and 17 months postnatal, VHWs continued monthly visits providing routine care. During these visits, VHWs informally encouraged participants to practise behaviours relevant to their treatment group, although formal modules were not implemented. At 18 months, a review module was implemented in all treatment groups before the mother completed the trial.

In the standard-of-care group, VHWs promoted exclusive breastfeeding to 6 months,^16^ advised on neonatal care, and promoted uptake of Ministry of Health and Child Care services, including antenatal care, immunisations, and family planning. In the WASH group, VHWs delivered all the standard-of-care messages, plus information about safe disposal of faeces, handwashing with soap at key times, protection of infants from geophagia and ingestion of animal faeces, chlorination of drinking water (especially for infants), and hygienic preparation of complementary food. Additionally, ventilated improved pit latrines were constructed and two handwashing stations were installed by community builders supervised by Ministry of Health and Child Care environmental health technicians within 6 weeks of enrolment. A plastic mat and play yard (North States, Minneapolis, MN, USA) were delivered to the home by a trial logistician at 2 and 6 months postnatal, respectively, and VHWs made monthly deliveries of soap from the time of the handwashing module (roughly 30 weeks antenatal) and chlorine from the time of the water treatment module (4 months postnatal) until 18 months postnatal. In the IYCF group, VHWs delivered all the standard-of-care messages plus information about the importance of nutrition for infant health, growth, and development; feeding nutrient-dense food and 20 g of a small-quantity lipid-based nutrient supplement^17^ (Nutriset, Malaumay, France) daily from age 6–18 months; processing foods to facilitate mastication and swallowing; feeding during illness; and dietary diversity. Participants also received monthly deliveries of 30 sachets of the small-quantity lipid-based nutrient supplement from VHWs from infant age 6 months to 18 months postnatal. In the combined group, participants received all standard-of-care, WASH, and IYCF interventions. Ventilated improved pit latrines were built for participants in the standard-of-care and IYCF groups after trial completion.

Research nurses made home visits at baseline (roughly 2 weeks after mothers provided consent), 32 weeks' gestation, and 1, 3, 6, 12, and 18 months post partum to assess maternal and household characteristics and trial outcomes. Intervention uptake was assessed at all visits and reported here as prespecified for the 12-month postnatal visit. At baseline, mothers' height, weight, mid-upper-arm circumference, and haemoglobin concentrations (with Hemocue, Ängelholm, Sweden) were measured. They were also tested for *Schistosoma haematobium* (by urinary microscopy) and HIV (via the rapid test algorithm). HIV-positive women were urged to seek immediate antenatal care for prevention of mother-to-child transmission. Other maternal and household characteristics assessed included dietary diversity, food insecurity, household wealth, and maternal capabilities.^18^

Infant birth date, weight, and delivery details were transcribed from health facility records. We provided Tanita BD-590 (Arlington Heights, IL, USA) infant scales to all health institutions in the study area and trained facility staff in use of the scales. Gestational age at delivery was calculated from the date of the mother's last menstrual period. Infant weight, length, head circumference, and mid-upper-arm circumference were measured at every postnatal visit (appendix). At the 18-month postnatal visit (ie, the trial endpoint), haemoglobin concentrations were measured, and length was calculated as the median of three measurements. Nurses were standardised against a gold standard anthropometrist by measuring non-study children from the community during a quality-control session held every 6 months. Infant diarrhoea (three or more loose or watery stools in 24 h), dysentery (stool with blood or mucus), and acute respiratory infection (fast or difficult breathing) were assessed by 7-day maternal recall at postnatal visits. Infants with acute malnutrition or illness were referred to clinics. At the 18-month postnatal visit (ie, the trial endpoint), mothers and infants were visited anywhere in the country. However, in view of the household-based nature of interventions, intermediate visits were done only when the mother was available in the household where she consented.

Serious adverse events and adverse events were ascertained during data collection visits and by VHWs, and were referred to a senior research nurse who collected details. Events were reviewed by the study physician (AJP) to establish relatedness to trial interventions before reporting them to responsible institutional review boards. An independent data safety and monitoring board comprising two physicians from Zimbabwe and a statistician from the UK reviewed interim adverse event data.

### Outcomes

We prespecified that primary trial inferences would be based on findings among infants born to mothers who were HIV negative during pregnancy. Primary outcomes were mean length-for-age Z score and haemoglobin concentration at 18 months (target age 76–80 weeks, allowable range 76–130 weeks) in all enrolled participants (intention-to-treat analysis). Secondary outcomes were mean weight-for-age Z scores, weight-for-length Z scores, mid-upper-arm circumference-for-age Z scores, and head circumference-for-age Z scores; the proportion of infants who were stunted (ie, length-for-age Z score less than −2), severely stunted (ie, length-for-age Z score less than −3), anaemic (ie, haemoglobin concentration <105 g/L), severely anaemic (ie, haemoglobin concentration <70 g/L), underweight (ie, weight-for-age Z scores less than −2), and wasted (ie, weight-for-height Z scores less than −2); mean prevalence of diarrhoea, dysentery, and acute respiratory infection based on 7-day maternal history at infant age 12 months and 18 months; and cumulative mortality up to age 18 months.

### Statistical analysis

The original sample size calculation was 4800 women to allow for 15% exclusion because of maternal HIV and 20% loss because of fetal and infant deaths, withdrawal, and loss to follow-up. Actual recruitment was 10% higher than the sample size calculation to ensure sufficient power for sensitivity analyses. With a minimum of 816 HIV-unexposed infants per group at 18 months, the trial was powered to detect a 0·2 difference in length-for-age Z scores, a reduction of 8 percentage points in stunting, and a 2·6 g/L shift in haemoglobin for the marginal effect of either intervention, with 90% power and type 1 error of 5%.^2^ This calculation was based on an assumed coefficient of variation of the true proportions of 0·43, and an effective loss of 33% of sample size because of variability in cluster size.

All analyses were done on an intention-to-treat basis at the child level. For primary analyses, we used generalised estimating equations that accounted for within-cluster correlation and contained two dummy variables representing the main effect of the IYCF intervention (the two IYCF groups compared with the two non-IYCF groups) and the WASH intervention (the two WASH groups compared with the two non-WASH groups), unadjusted for other covariates, with an exchangeable working correlation structure.^11^ Although the study was not powered to detect a statistical interaction between the two interventions, we estimated these interactions for each outcome. When the interaction was significant (ie, p<0·05 according to the Wald test) or had a sizeable point estimate (ie, relative risk [RR] >2 or <0·5 when comparing ratio-of-ratios, or difference-of-differences >0·25 SDs when comparing continuous outcomes), results were based on a regression model with three dummy variables to represent IYCF, WASH, and IYCF plus WASH compared with standard of care instead of the model of two terms. In adjusted analyses we controlled for prespecified baseline covariates, which were initially assessed in bivariate analyses to identify those with an important association with the outcome (ie, p<0·2 or RR >2·0 or <0·5 for dichotomous outcomes, and p<0·2 or difference >0·25 SDs for continuous outcomes). Selected covariates were entered in a multivariable regression model; a forward stepwise selection procedure was implemented with p<0·2 to enter. A log-binomial specification was used to facilitate estimation of RRs. Depending on the analysis, other methods for comparison of groups while accounting for within-cluster correlation included multinomial and ordinal regression models with robust variance estimation, and Somers' D for medians.

In a per-protocol analysis, we examined the effect of the interventions when behaviour-change modules were delivered at high fidelity (which was predefined for the IYCF plus WASH group as receiving ten core modules and for the other study groups as receiving all modules scheduled at the same timepoints when IYCF plus WASH core modules were delivered). Several sensitivity analyses were prespecified: exclusion of mothers enrolled before Nov 1, 2013, to account for initial delays in latrine construction; exclusion of children born to women who were HIV negative during pregnancy but HIV positive at 18 months; and restriction of analyses to children in whom primary outcomes were measured during tight infant age windows (ie, at age 76–80 weeks and 76–100 weeks). A prespecified subgroup analysis of primary outcomes by infant sex was planned if the interaction terms were significant.

We used Stata (version 14.1) for all analyses. This trial is registered with ClinicalTrials.gov, number NCT01824940.

### Role of the funding source

The study funders approved the trial design, but had no roles in data collection, analysis, or interpretation, or writing of the report. The corresponding author had full access to all study data and had final responsibility for the decision to submit for publication.

## Results

5280 pregnant women were enrolled from 211 clusters at a median gestational age of 12 (IQR 9–16) weeks (figure). During the antenatal period, 11 women were excluded and one woman was added to the analysis to correct for enrolment errors (figure). 139 (3%) women left the trial or were lost to follow-up, four (<1%) died, 249 (5%) miscarried, and 101 (2%) had stillbirths. 726 (14%) women tested positive for HIV and 114 (2%) had unknown HIV statuses during pregnancy, and were thus excluded from the analysis (outcomes for these infants will be reported separately). Thus 3989 infants were born alive to 3937 HIV-negative women and were included in our analysis. During the postnatal period, 198 (5%) infants died. 3686 (97%) of the remaining 3791 liveborn infants were assessed at the 18-month endpoint (5 [<1%] left the trial and 100 [3%] were lost to follow-up or moved outside Zimbabwe).

**Figure:**
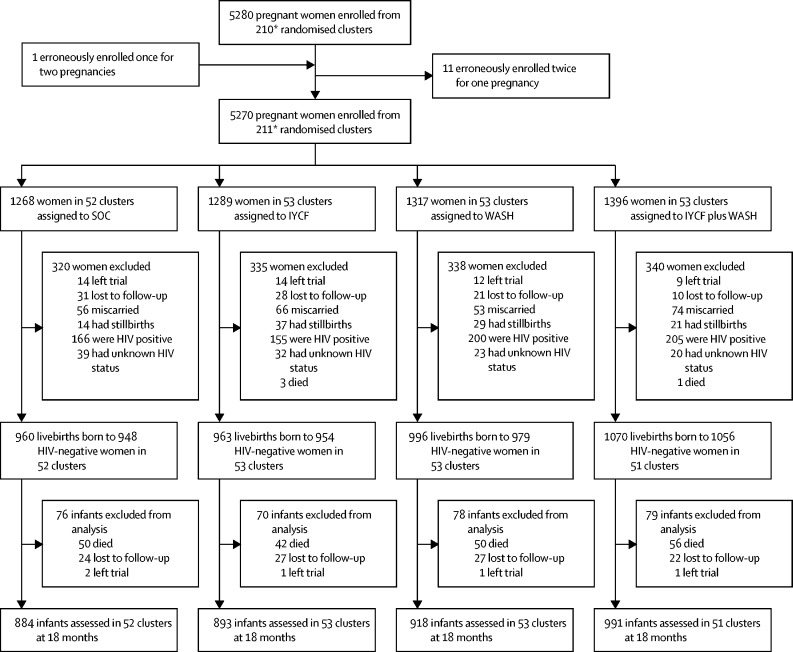
Trial profile SOC=standard of care. IYCF=infant and young child feeding. WASH=water, sanitation, and hygiene. *212 clusters were randomly assigned, 53 in each of the four trial groups. After randomisation, one cluster was excluded because it was in an urban area, one was excluded because the village health workers covering it mainly had clients outside the study area, and two more were merged on the basis of subsequent data for village health worker coverage. Three new cluster designations were created because of anomalies in the original mapping. For two of these clusters, the trial group was clear; the third contained areas that were in two trial groups, and was assigned to the under-represented group, resulting in 53 clusters in each group. All these changes occurred before enrolment began. When enrolment was completed, however, no women were enrolled in one cluster in the SOC group and thus 211 clusters were available for analysis.

At baseline, nearly half of all household members practised open defecation and about a third of households had an improved latrine (table 1). Very few had electricity from the grid but two-thirds had a solar panel (typically for phone charging; table 1). Water access was poor: nearly 40% of households obtained drinking water from unimproved sources, and about 40% had a return-trip walk to their water source of greater than 30 min (data not shown). The mean volume collected was 9·5 L per person per day (table 1). Walking time to water was not associated with volume collected (data not shown). Mothers had completed a median of more than 9 years of schooling, were mostly married, and roughly 10% were infected with *S haematobium* (table 1). Mean infant birthweight was 3·1 kg (SD 0·5), and nearly 90% were born via institutional deliveries (table 1). The frequency of open defecation was higher—and wealth index scores, rates of employment and latrine ownership, and diet quality were lower—in mothers in the standard-of-care group than in those in the other groups. Other maternal and household baseline characteristics and all infant birth characteristics were similar across treatment groups (table 1).

**Table 1:** Maternal, household, and infant baseline characteristics of HIV-negative mothers and their liveborn infants

				**Standard of care**	**Infant and young child feeding**	**Water, sanitation, and hygiene**	**Water, sanitation, and hygiene plus infant and young child feeding**
Mothers				948	954	979	1056
Infants				960	963	996	1070
Mothers completing baseline visit				866	867	943	1013
Household characteristics							
	Median number of occupants (IQR)			5 (3–6)	5 (4–6)	5 (3–6)	5 (3–6)
	Wealth quintile^19^						
		1 (lowest)		189/858 (22%)	132/861 (15%)	189/935 (20%)	170/1005 (17%)
		2		163/858 (19%)	156/861 (18%)	181/935 (19%)	206/1005 (20%)
		3		171/858 (20%)	187/861 (22%)	178/935 (19%)	207/1005 (21%)
		4		169/858 (20%)	188/861 (22%)	202/935 (22%)	208/1005 (21%)
		5 (highest)		166/858 (19%)	198/861 (23%)	185/935 (20%)	214/1005 (21%)
	Electricity						
		Power grid		26/855 (3%)	33/857 (4%)	26/938 (3%)	15/1006 (1%)
		Other power source					
			Solar	548/855 (64%)	584/858 (68%)	653/938 (70%)	698/1006 (69%)
			Generator	22/855 (3%)	34/858 (4%)	31/938 (3%)	31/1006 (3%)
			No electricity	285/855 (33%)	240/858 (28%)	254/938 (27%)	277/1006 (28%)
	Sanitation						
		Household members defecate in the open		1924/3602 (53%)	1854/3900 (48%)	1916/4009 (48%)	1988/4354 (46%)
		Any latrine at household		280/852 (33%)	346/850 (41%)	331/917 (36%)	367/987 (37%)
		Improved latrine at household		245/852 (29%)	301/849 (35%)	293/914 (32%)	318/986 (32%)
		Improved latrine with well trodden path not shared with other households and not used for storage		178/829 (21%)	233/817 (29%)	229/886 (26%)	250/952 (26%)
	Water						
		Main source of household drinking water improved		529/855 (62%)	558/854 (65%)	567/923 (61%)	639/993 (64%)
		Treated drinking water to make safer		118/840 (14%)	108/836 (13%)	109/912 (12%)	114/980 (12%)
		Median one-way walk time to fetch water (IQR), min		10 (5–20)	10 (5–18)	10 (5–20)	10 (5–20)
		Mean water volume collected per person in past 24 h (SD), L		9·5 (10·5)	9·6 (8·6)	9·9 (16·6)	9·6 (10·0)
	Hygiene						
		Handwashing station at household		37/796 (5%)	20/812 (2%)	121/885 (14%)	130/940 (14%)
		Handwashing station with water and rubbing agent		10/793 (1%)	1/812 (<1%)	8/884 (1%)	10/938 (1%)
		Improved floor*		442/845 (52%)	481/851 (57%)	516/921 (56%)	557/990 (56%)
		Median number of chickens (IQR)		6 (2–10)	7 (2–12)	6 (2–10)	5 (2–10)
		Livestock observed inside home		324/859 (38%)	345/861 (40%)	341/931 (37%)	363/1003 (36%)
		Faeces observed in the yard		271/855 (32%)	323/857 (38%)	274/928 (30%)	268/995 (27%)
	Diet quality and food security						
		Household meets minimum Diet Diversity Score^20^		292/769 (38%)	326/744 (44%)	323/833 (39%)	353/878 (40%)
		Median Coping Strategies Index score^21^ (IQR)		1 (0–7)	0 (0–6)	1 (0–7)	1 (0–7)
Maternal characteristics							
	Mean age (SD), years			25·4 (8·5)	25·7 (7·6)	25·6 (8·5)	25·9 (8·1)
	Mean height (SD), cm			156·0 (6·1)	160·1 (5·9)	159·5 (8·0)	159·6 (10·2)
	Mean mid-upper-arm circumference (SD), cm			26·3 (3·2)	26·5 (3·2)	26·4 (3·6)	26·5 (3·3)
	Positive microscopy for *Schistosoma haematobium*			78/824 (9%)	80/820 (10%)	116/878 (13%)	104/958 (11%)
	Mean years of schooling completed (SD)			9·6 (2·2)	9·7 (2·8)	9·5 (2·0)	9·6 (2·5)
	Median parity (IQR)			2 (1–3)	2 (1–3)	2 (1–3)	2 (1–3)
	Married			850/894 (95%)	852/892 (96%)	886/933 (95%)	958/998 (10%)
	Employed			53/857 (6%)	81/857 (9%)	91/936 (10%)	86/1006 (9%)
	Religion						
		Apostolic		459/899 (51%)	395/903 (44%)	440/939 (47%)	469/1004 (47%)
		Other Christian		383/899 (43%)	444/903 (49%)	410/939 (44%)	447/1004 (45%)
		Other		57/899 (6%)	64/903 (7%)	89/939 (9%)	88/1004 (9%)
Infant characteristics							
	Female sex			474/959 (49%)	472/958 (49%)	487/995 (49%)	529/1062 (50%)
	Mean birthweight (SD), kg			3·1 (0·6)	3·10 (0·49)	3·09 (0·54)	3·10 (0·50)
	Birthweight <2500 g			79/855 (9%)	76/864 (9%)	86/892 (10%)	84/962 (9%)
	Institutional delivery			752/855 (88%)	761/861 (88%)	794/892 (89%)	854/948 (90%)
	Vaginal delivery			814/874 (93%)	812/870 (93%)	833/904 (92%)	897/967 (93%)

Baseline variables are presented for mothers who had livebirths. Maternal and household data were collected about 2 weeks after consent was recorded (at roughly 14 weeks' gestation). This gap created opportunity for loss to follow-up between consent and baseline; thus, the number of mothers completing baseline visit is less than the number of mothers with livebirths. Baseline for infants was birth. Data are n or n (%), unless otherwise specified.

* Improved floor defined as concrete, brick, cement, or tile; unimproved floor defined as mud, earth, sand, or dung.

Fidelity of intervention delivery was high (table 2). Among households in the WASH groups, more than 98% received ventilated improved pit latrines and handwashing stations, more than 92% received play mats and play yards, and nearly 80% received 80% or more of the planned deliveries of soap and chlorine solution (table 2). Among households in the IYCF groups, 79% received ≥80% of planned deliveries of the small-quantity lipid-based nutrient supplement (table 2). Among households in the WASH groups and IYCF groups, mothers received a median of 15 (IQR 13–15) of the 15 intervention visits scheduled between enrolment and 12 months postnatal. Thus, the intervention dose (ie, frequency of contact) was about once per 5 weeks.

**Table 2:** Intervention delivery and participant uptake by treatment group

		**Data source**	**Standard of care**	**IYCF**	**WASH**	**IYCF plus WASH**	**Combined WASH**	**Non-WASH**	p value*	**Combined IYCF**	**Non-IYCF**	p value*
**Fidelity of intervention delivery**												
Children with 18-month outcomes (on whom inferences are based), n		Trial logs	884	893	918	991	1909	1777		1884	1802	
WASH supplies												
	SHINE-installed ventilated improved pit latrine	Trial logs	NA	NA	901/918 (98%)	974/991 (98%)	1875/1909 (98%)	NA	..	NA	NA	..
	Two handwashing stations (Tippy Taps) delivered	Trial logs	NA	NA	912/918 (99%)	986/991 (99%)	1898/1909 (99%)	NA	..	NA	NA	..
	Baby mat delivered	Trial logs	NA	NA	859/918 (94%)	942/991 (95%)	1801/1909 (94%)	NA	..	NA	NA	..
	Play yard delivered	Trial logs	NA	NA	847/918 (92%)	926/991 (93%)	1773/1909 (93%)	NA	..	NA	NA	..
	Median liquid soap deliveries (IQR)†	Trial logs	NA	NA	20 (16–20)	20 (18–20)	20 (17–20)	NA	..	NA	NA	..
	Received ≥80% of expected soap deliveries	Trial logs	NA	NA	709/918 (77%)	801/991 (81%)	1510/1909 (79%)	NA	..	NA	NA	..
	Median Water Guard deliveries (IQR)‡	Trial logs	NA	NA	15 (13–15)	15 (14–15)	15 (14–15)	NA	..	NA	NA	..
	Received ≥80% of expected Water Guard deliveries	Trial logs	NA	NA	724/918 (79%)	799/991 (81%)	47232/52656 (80%)	NA	..	NA	NA	..
IYCF supplies												
	Median deliveries of small-quantity lipid-based nutrient supplement (IQR)§	Trial logs	NA	13 (12–13)	NA	13 (12–13)	NA	NA	..	13 (12–13)	NA	..
	Received ≥11 (80% of expected) deliveries of small-quantity lipid-based nutrient supplement	Trial logs	NA	695/893 (78%)	NA	790/991 (80%)	NA	NA	..	1485/1884 (79%)	NA	..
Behaviour change modules												
	Median intervention modules (IQR)‡	Village health worker report	15 (13–15)	15 (13–15)	14 (13–15)	15 (14–15)	15 (13–15)	15 (13–15)	0·321	15 (13–15)	15 (13–15)	0·0009
	Completed intervention modules	Village health worker report	14 664/16 523 (89%)	19 673/22 162 (88·8%)	20 636/23 237 (89%)	26 596/29 419 (90%)	47 232/52 656 (90%)	34 337/38 685 (89%)	0·321	46 269/51 581 (90%%)	35 300/39 760 (89%)	0·347
**Participant uptake of promoted behaviours at the 12-month visit**												
Mothers with outcomes at 12 months and 18 months, n		Trial logs	675	702	652	731	1383	1377	..	1433	1327	..
Children with outcomes at 12 months and 18 months, n		Trial logs	682	706	662	741	1403	1388	..	1447	1344	..
WASH behaviours												
	Household members who defecate in the open	Maternal report	1088/2407 (45%)	1057/2789 (38%)	27/2917 (1%)	23/3359 (1%)	50/6276 (1%)	50/6276 (1%)	<0·0001	NA	NA	..
	Any latrine at household	Observation	214/665 (32%)	291/683 (43%)	642/648 (99%)	725/727 (>99%)	1367/1375 (99%)	505/1348 (37%)	<0·0001	NA	NA	..
	Improved latrine at household	Observation	185/664 (28%)	246/683 (36%)	642/648 (99%)	723/727 (>99%)	1365/1375 (99%)	431/1347 (32%)	<0·0001	NA	NA	..
	Improved latrine with well trodden path not shared with other households and not used for storage	Observation and maternal report	144/662 (22%)	182/682 (27%)	557/647 (86%)	624/726 (86%)	1181/1373 (86%)	326/1344 (24%)	<0·0001	NA	NA	..
	Handwashing station at household	Observation	38/640 (6%)	41/663 (6%)	641/653 (98%)	721/734 (98%)	1362/1387 (98%)	79/1303 (6%)	<0·0001	NA	NA	..
	Handwashing station with water and rubbing agent at household	Observation	6 (3%)	13/654 (2%)	513/615 (83%)	560/669 (84%)	1073/1284 (84%)	31/1286 (2%)	<0·0001	NA	NA	..
	Ever treats drinking water to make safer	Maternal report	90/667 (13%)	77/684 (11%)	567/650 (87%)	630/727 (87%)	1197/1377 (87%)	167/1351 (12%)	<0·0001	NA	NA	..
	Disposes water from cleaning infant nappies with faeces in a latrine	Maternal report	185/660 (28%	239/671 (36%)	478/634 (75%)	558/711 (78%)	1036/1345 (77%)	424/1331 (32%)	<0·0001	NA	NA	..
	Play space is visibly clean	Observation	NA	NA	563/615 (92%)	638/688 (93%)	1201/1303 (92%)	NA	NA	NA	NA	..
	Child ever observed to eat soil	Maternal report	518/663 (78%)	470/691 (68%)	166/645 (26%)	199/725 (27%)	365/1370 (27%)	988/1354 (73%)	<0·0001	NA	NA	..
	Child ever observed to eat chicken faeces	Maternal report	152/663 (23%)	131/689 (19%)	19/646 (3%)	17/724 (2%)	36/1370 (3%)	283/1352 (21%)	<0·0001	NA	NA	..
	IYCF behaviours											
	Child still breastfeeding	Maternal report	655/677 (97%)	675/695 (97%)	634/653 (97%)	703/734 (96%)	NA	NA	−	1378/1429 (96%)	1289/1330 (97%)	0·494
	Mother reports correct ways to feed child during and after illness	Maternal report	424/673 (63%)	485/690 (70%)	393/647 (61%)	499/729 (68%)	NA	NA	−	984/1419 (69%)	817/1320 (62%)	<0·0001
	Infant diet met minimum dietary diversity in past 24 h	Maternal report	343/656 (52%)	460/670 (69%)	338/627 (54%)	496/700 (71%)	NA	NA	−	956/1370 (70%)	681/1283 (53%)	<0·0001
	Infant consumed iron-rich food in past 24 h	Maternal report	333/664 (50%)	665/692 (96%)	309/643 (48%)	697/729 (96%)	NA	NA	−	1362/1421 (96%)	642/1307 (49%)	<0·0001
	Infant consumed animal source food in past 24 h	Maternal report	419/667 (63%)	483/688 (70%)	394/640 (62%)	508/719 (71%)	NA	NA	−	991/1407 (70%)	813/1307 (62%)	<0·0001
	Infant consumed vitamin-A-rich food in past 24 h	Maternal report	469/668 (70%)	540/688 (78%)	434/643 (67%)	571/719 (79%)	NA	NA	−	1111/1407 (79%)	903/1311 (69%)	<0·0001
	Nutributter consumed in past 24 h	Maternal report	NA	634/668 (95%)	NA	645/714 (90%)	NA	NA	−	1111/1407 (79%)	NA	..

Data are n/N (%), unless otherwise specified. The denominator for indictors of fidelity of intervention delivery are the number of children who provided 18-month outcomes, because inferences of trial outcomes are based on these children. The denominator for indicators of participant uptake of promoted behaviours at the 12-month visit are the number of women (for household-level indicators) and children (for child-level indicators) who provided 12-month and 18-month outcomes. Village health workers were scheduled to visit households monthly to deliver 30 sachets of a small-quantity lipid-based nutrient supplement (sufficient to provide 20 g per day), 1 L of liquid soap, and 150 mL (one bottle) of Water Guard for families of less than five people (two bottles for families of five or more people). The combined WASH group comprised the two WASH-containing groups, whereas the non-WASH group comprised the two groups not including WASH. The combined IYCF group comprised the two IYCF-containing groups, whereas the non-IYCF group comprised the two groups not including IYCF. IYCF=infant and young child feeding. WASH=water, sanitation, and hygiene. SHINE=Sanitation, Hygiene, Infant Nutrition Efficacy trial. NA=not applicable.

* p values were adjusted for clustering effect; depending on the variable type, xtgee, multinomial, ordinal regression models with robust variance estimation, and Somers' D for medians, were used for comparing arms while accounting for within-cluster correlation.

† Maximum of 20 deliveries.

‡ Maximum of 15 deliveries.

§ Maximum of 13 deliveries.

Intervention uptake was assessed at 12 months, when 74% of the women were available for the visit (table 2). Women assessed at 12 months were, on average, older, slightly wealthier, more likely to be married and have a diverse diet, and had higher parity than women not assessed at the 12-month visit (appendix). Baseline indicators of sanitation, water, and hygiene were similar (appendix).

At the 12 months post-partum visit, the frequency of open defecation among household members in the WASH groups was 1% compared with 41% in non-WASH groups (table 2). Nearly all households in WASH groups had an improved latrine, and in 86% of households the latrine had a well trodden path and was not being used for storage (compared with 24% in non-WASH groups). 84% of households in the WASH groups had a handwashing station with observed soap or rubbing agent and water compared with 2% of households in non-WASH groups (table 2). 1197 (87%) of 1372 women in the WASH groups reported that they usually treated their drinking water. However, too few samples of water were tested for free chlorine to objectively validate water chlorination. Of the 752 water samples from WASH households that were tested at 12 months, only 438 (58%) had more than 0·1 parts per million of free chlorine. Compared with infants in the non-IYCF groups, a higher proportion of those in the IYCF groups had diets that met minimum dietary diversity and had consumed animal-source, iron-rich, and vitamin-A-rich foods in the previous 24 h (table 2). 93% of children in the IYCF groups consumed the small-quantity lipid-based nutrient supplement in the previous 24 h (table 2).

At the 18-month visit, median child age was 18·0 months (IQR 17·8–18·8) and did not differ significantly across treatment groups (data not shown). There was no statistical interaction between the two treatments for any outcome at 18 months, so the main effects of the IYCF and WASH interventions are presented. All follow-up was completed by July 31, 2017. At 18 months, the mean length-for-age Z score was 0·16 (95% CI 0·08–0·23) higher and mean haemoglobin concentration was 2·0 g/L (1·28–2·79) higher in children in the IYCF groups than in those in the non-IYCF groups (table 3). These differences were slightly attenuated in adjusted analyses (table 3). The IYCF intervention reduced stunting by 7·2 percentage points (95% CI 4·3–10·2)—620 (35%) of 1792 children in the non-IYCF groups *vs* 514 (27%) of 1879 children in the IYCF groups. The IYCF intervention also reduced anaemia by 3·5 percentage points (1·3–5·6)—245 (14%) of 1759 children in the non-IYCF groups *vs* 194 (11%) of 1845 children in the IYCF groups—and significantly increased mean weight-for-age, weight-for-height, and head-circumference-for-age Z scores (Table 3, Table 4) compared with the non-IYCF interventions.

**Table 3:** Effect of WASH and IYCF interventions on infant growth and haemoglobin concentrations at age 18 months (primary and secondary continuous outcomes)

	**Effects by individual treatment group**		**Main effects combining groups**			**Unadjusted difference (95% CI)**	**p value**	Adjusted difference*(95% CI)	**p value**
	n	Mean (SD)	Treatment group	n	Mean (SD)*				
**Length-for-age Z score**									
SOC	878	−1·57 (1·08)	No IYCF	1792	−1·59 (1·08)	Ref	..	Ref	..
IYCF	891	−1·47 (1·06)	IYCF	1879	−1·44 (1·06)	0·16 (0·08 to 0·23)	<0·0001	0·14 (0·07 to 0·21)	<0·0001
WASH	914	−1·61 (1·07)	No WASH	1769	−1·52 (1·07)	Ref	..	Ref	..
IYCF plus WASH	988	−1·41 (1·06)	WASH	1902	−1·50 (1·07)	0·02 (−0·06 to 0·09)	0·698	0·06 (−0·01 to 0·12)	0·119
**Haemoglobin (g/dL)**									
SOC	866	116·5 (11·3)	No IYCF	1759	116·3 (11·8)	Ref	..	Ref	..
IYCF	882	118·4 (11·2)	IYCF	1845	118·3 (11·5)	2·03 (1·28 to 2·79)	<0·0001	1·94 (1·22 to 2·67)	<0·0001
WASH	893	116·1 (12·4)	No WASH	1748	117·5 (11·3)	Ref	..	Ref	..
IYCF plus WASH	963	118·3 (11·8)	WASH	1856	117·2 (12·1)	−0·28 (−1·04 to 0·48)	0·471	−0·60 (−1·37 to 0·17)	0·128
**Weight-for-age Z score**									
SOC	875	−0·72 (1·02)	No IYCF	1785	−0·75 (1·02)	Ref	..	Ref	..
IYCF	888	−0·66 (1·02)	IYCF	1871	−0·62 (0·99)	0·13 (0·07 to 0·20)	<0·0001	0·13 (0·07 to 0·19)	<0·0001
WASH	910	−0·78 (1·02)	No WASH	1763	−0·69 (1·02)	Ref	..	Ref	..
IYCF plus WASH	983	−0·59 (0·97)	WASH	1893	−0·68 (1·00)	0·00 (−0·06 to 0·07)	0·911	0·00 (−0·06 to 0·06)	0·971
**Weight-for-height Z score**									
SOC	875	0·05 (1·07)	No IYCF	1782	0·02 (1·05)	Ref	..	Ref	..
IYCF	888	0·06 (1·11)	IYCF	1870	0·09 (1·07)	0·08 (0·00 to 0·15)	0·036	0·08 (0·02 to 0·15)	0·016
WASH	907	−0·01 (1·04)	No WASH	1763	0·06 (1·09)	Ref	..	Ref	..
IYCF plus WASH	982	0·11 (1·04)	WASH	1889	0·05 (1·04)	−0·01 (−0·08 to 0·07)	0·875	−0·04 (−0·11 to 0·03)	0·257
**Mid-upper-arm circumference Z score**									
SOC	870	0·03 (0·90)	No IYCF	1779	0·01 (0·92)	Ref	..	Ref	..
IYCF	889	0·05 (0·87)	IYCF	1871	0·07 (0·84)	0·07 (0·01 to 0·13)	0·033	0·07 (0·01 to 0·14)	0·018
WASH	909	−0·01 (0·92)	No WASH	1759	0·04 (0·88)	Ref	..	Ref	..
IYCF plus WASH	982	0·09 (0·82)	WASH	1891	0·04 (0·88)	0·00 (−0·06 to 0·06)	0·999	0·01 (−0·05 to 0·07)	0·745
**Head-circumference-for-age Z score**									
SOC	872	−0·26 (1·08)	No IYCF	1778	−0·26 (1·08)	Ref	..	Ref	..
IYCF	885	−0·23 (1·07)	IYCF	1868	−0·19 (1·06)	0·07 (0·00 to 0·14)	0·043	0·06 (0·00 to 0·13)	0·053
WASH	906	−0·27 (1·09)	No WASH	1757	−0·24 (1·07)	Ref	..	Ref	..
IYCF plus WASH	983	−0·16 (1·06)	WASH	1889	−0·21 (1·08)	0·03 (−0·04 to 0·10)	0·372	0·08 (0·01 to 0·15)	0·018

SOC=standard of care. IYCF=infant and young child feeding. Ref=reference. WASH=water, sanitation, and hygiene.

* Prespecified baseline variables considered for inclusion in adjusted analyses were maternal age, mid-upper-arm circumference, years of schooling, marital status, employment, religion, maternal capabilities, haemoglobin concentration, household Coping Strategy Index, proportion of household members practising open defecation, faeces observed in yard, household floor type, time to fetch drinking water, chicken ownership, livestock observed inside house, number of household occupants, wealth index quintile, infant low versus normal birthweight, infant sex, and preterm birth; the study factors data collector and calendar year of enrolment were also considered for inclusion.

**Table 4:** Effect of WASH and IYCF interventions on infant growth and haemoglobin concentrations at age 18 months (secondary dichotomous outcomes)

	**Effects by individual treatment group**		**Main effects combining groups**			**Unadjusted RR (95% CI)**	**p value**	Adjusted RR*(95% CI)	**p value**
	n	Prevalence (%)	Treatment group	n	Prevalence (%)				
**Stunting (length-for-age Z score less than −2·0)**									
SOC	878	292 (33%)	No IYCF	1792	620 (35%)	Ref	..	Ref	..
IYCF	891	249 (28·0%)	IYCF	1879	514 (27%)	0·79 (0·72–0·87)	<0·0001	0·80 (0·73–0·88)	<0·0001
WASH	914	328 (36%)	No WASH	1769	541 (31%)	Ref	..	Ref	..
IYCF plus WASH	988	265 (27%)	WASH	1902	593 (31%)	1·03 (0·93–1·13)	0·596	0·99 (0·90–1·09)	0·818
**Severe stunting (length-for-age Z score less than −3·0)**									
SOC	878	74 (8%)	No IYCF	1792	160 (9%)	Ref	..	Ref	..
IYCF	891	72 (8%)	IYCF	1879	139 (7%)	0·83 (0·66–1·04)	0·109	0·85 (0·67–1·07)	0·173
WASH	914	86 (9%)	No WASH	1769	146 (8%)	Ref	..	Ref	..
IYCF plus WASH	988	67 (7%)	WASH	1902	153 (8%)	0·99 (0·78–1·24)	0·908	0·96 (0·75–1·23)	0·769
**Anaemia (haemoglobin <105 g/L)**									
SOC	866	117 (14%)	No IYCF	1759	245 (14%)	Ref	..	Ref	..
IYCF	882	81 (9%)	IYCF	1845	193 (10%)	0·75 (0·62–0·90)	0·003	0·76 (0·63–0·92)	0·004
WASH	893	128 (14%)	No WASH	1748	198 (11%)	Ref	..	Ref	..
IYCF plus WASH	963	112 (12%)	WASH	1856	240 (13%)	1·14 (0·95–1·36)	0·151	1·13 (0·93–1·37)	0·235
**Severe anaemia (haemoglobin <70 g/L)**									
SOC	866	3 (<1%)	No IYCF	1759	5 (<1%)	Ref	..	..†	..
IYCF	882	0 (0%)	IYCF	1845	1 (<1%)	0·19 (0·02–1·62)	0·129	..	..
WASH	893	2 (<1%)	No WASH	1748	3 (<1%)	Ref		..	..
IYCF plus WASH	963	1 (<1%)	WASH	1856	3 (<1%)	0·96 (0·20–4·71)	0·959	..	..
**Underweight (weight-for-age Z score less than −2·0)**									
SOC	875	83 (9%)	No IYCF	1785	189 (11%)	Ref	..	Ref	..
IYCF	888	74 (8%)	IYCF	1871	147 (8%)	0·74 (0·60–0·91)	0·005	0·76 (0·62–0·94)	0·010
WASH	910	106 (12%)	No WASH	1765	157 (9%)	Ref	..	Ref	..
IYCF plus WASH	983	73 (7%)	WASH	1893	179 (9%)	1·07 (0·87–1·31)	0·539	1·08 (0·87–1·34)	0·464
**Wasted (weight-for-height Z score less than −2·0)**									
SOC	875	25 (3%)	No IYCF	1782	48 (3%)	Ref	..	Ref	..
IYCF	888	24 (3%)	IYCF	1870	43 (2%)	0·86 (0·57–1·29)	0·459	0·83 (0·55–1·27)	0·381
WASH	907	23 (3%)	No WASH	1763	49 (3%)	Ref	..	Ref	..
IYCF plus WASH	982	19 (2%)	WASH	1889	42 (2%)	0·80 (0·53–1·21)	0·291	0·88 (0·55–1·39)	0·571

RR=relative risk. SOC=standard of care. IYCF=infant and young child feeding. Ref=reference. WASH=water, sanitation, and hygiene.

* Prespecified baseline variables considered for inclusion in adjusted analyses were maternal age, mid-upper-arm circumference, years of schooling, marital status, employment, religion, maternal capabilities, haemoglobin concentration, household Coping Strategy Index, proportion of household members practising open defecation, faeces observed in yard, household floor type, time to fetch drinking water, chicken ownership, livestock observed inside house, number of household occupants, wealth index quintile, infant low versus normal birthweight, infant sex, and preterm birth; the study factors data collector and calendar year of enrolment were also considered for inclusion.

† Insufficient data to run a regression model.

The WASH intervention had no effect on the mean infant length-for-age Z score or mean haemoglobin concentration compared with the non-WASH interventions (table 3). The WASH interventions had no significant effects on any other growth measurements except for mean head-circumference-for-age Z scores in adjusted analyses (table 3); however, this effect was driven entirely by the IYCF plus WASH group. Coefficient of variation estimates from random-effects models were 0·012 for stunting and 0·12 for anaemia, and 0·16 and 0, respectively, by method-of-moments estimation.

At the 12-month visit, there was a significant statistical interaction between the IYCF and WASH interventions for 7-day prevalence of diarrhoea and acute respiratory infection, so the IYCF, WASH, and IYCF plus WASH groups were each compared with the standard-of-care group (table 5). The 7-day prevalence of diarrhoea was 9% in the standard-of-care group, 13% in the IYCF group, 12% in the WASH group, and 10% in the IYCF plus WASH group (table 5). The prevalence of diarrhoea was 37% (95% CI 4–80) higher in the IYCF group than in the standard-of-care group (p=0·03). No significant differences were noted between the standard-of-care group and the IYCF group, the WASH group, or the IYCF plus WASH group in the prevalence of acute respiratory infection (table 5). There were only eight cases of dysentery overall (one in the standard-of-care group, three in the WASH group, and four in the IYCF plus WASH group).

**Table 5:** Effect of IYCF and WASH interventions on diarrhoea and acute respiratory infection at age 12 months

	**n**	**Prevalence**	**Difference *vs* SOC (95% CI)**			
			Unadjusted	p value	Adjusted*	p value
**Diarrhoea**						
SOC	678	62 (9%)	Ref	..	Ref	..
IYCF	696	87 (13%)	1·37 (1·04–1·80)	0·027	1·32 (1·00–1·75)	0·054
WASH	666	77 (12%)	1·26 (0·92–1·71)	0·151	1·18 (0·87–1·61)	0·292
IYCF plus WASH	735	76 (10%)	1·13 (0·84–1·53)	0·422	1·05 (0·79–1·40)	0·716
**Acute respiratory infection**						
SOC	676	6 (1%)	Ref	..	Ref	..
IYCF	694	2 (<1%)	0·32 (0·07–1·52)	0·154	0·34 (0·07–1·70)	0·193
WASH	662	8 (1%)	1·36 (0·51–3·63)	0·539	1·75 (0·62–4·90)	0·289
IYCF plus WASH	735	7 (1%)	1·07 (0·35–3·26)	0·899	1·38 (0·42–4·47)	0·595

SOC=standard of care. Ref=reference. IYCF=infant and young child feeding. WASH=water, sanitation, and hygiene.

* Prespecified baseline variables considered for inclusion in adjusted analyses were maternal age, mid-upper-arm circumference, years of schooling, marital status, employment, religion, maternal capabilities, haemoglobin concentration, household Coping Strategy Index, proportion of household members practising open defecation, faeces observed in yard, household floor type, time to fetch drinking water, chicken ownership, livestock observed inside house, number of household occupants, wealth index quintile, infant low versus normal birthweight, infant sex, and preterm birth; the study factors data collector and calendar year of enrolment were also considered for inclusion.

At 18 months, there was no interaction between the two treatments, so the main effects of the two interventions are presented (table 6). The prevalence of diarrhoea did not differ between the IYCF groups and non-IYCF groups, but was 28% higher in the WASH groups than in the non-WASH groups—a difference was significant in unadjusted but not adjusted analyses (table 6). Neither IYCF nor WASH significantly affected the prevalence of acute respiratory infection (table 6), and only ten cases of dysentery (two in the standard-of-care group, three in the IYCF group, two in the WASH group, and three in the IYCF plus WASH group) were recorded.

**Table 6:** Effect of IYCF and WASH interventions on diarrhoea, acute respiratory infection, and mortality at age 18 months

	**n**	**Prevalence**	**Main effects combining groups**			**Unadjusted difference (95% CI)**	**p value**	Adjusted*difference (95% CI)	**p value**
			Treatment group	n	Prevalence				
**Diarrhoea**									
SOC	874	83 (9%)	No IYCF	1784	176 (10%)	Ref	..	Ref	..
IYCF	883	65 (7%)	IYCF	1866	175 (9%)	0·95 (0·77–1·16)	0·585	0·97 (0·79–1·19)	0·750
WASH	910	93 (10%)	No WASH	1757	148 (8%)	Ref	..	Ref	..
IYCF plus WASH	983	110 (11%)	WASH	1893	203 (11%)	1·28 (1·04–1·57)	0·020	1·15 (0·93–1·41)	0·191
**Acute respiratory infection**									
SOC	875	6 (1%)	No IYCF	1785	11 (1%)	Ref	..	Ref	..
IYCF	879	4 (<1%)	IYCF	1856	9 (<1%)	0·77 (0·28–2·13)	0·617	0·76 (0·28–2·03)	0·582
WASH	910	5 (1%)	No WASH	1754	10 (1%)	Ref	..	Ref	..
IYCF plus WASH	977	5 (1%)	WASH	1887	10 (1%)	0·96 (0·36–2·55)	0·930	1·29 (0·48–3·43)	0·611
**Death**									
SOC	959	50 (5%)	No IYCF	1954	99 (5%)	Ref	..	Ref	..
IYCF	958	40 (4%)	IYCF	2020	92 (5%)	0·88 (0·66–1·18)	0·406	0·87 (0·65–1·18)	0·376
WASH	995	49 (5%)	No WASH	1917	90 (5%)	Ref	..	Ref	..
IYCF plus WASH	1062	52 (5%)	WASH	2057	101 (5%)	1·04 (0·78–1·39)	0·790	0·96 (0·72–1·30)	0·808

SOC=standard of care. IYCF=infant and young child feeding. Ref=reference. WASH=water, sanitation, and hygiene.

* Prespecified baseline variables considered for inclusion in adjusted analyses were maternal age, mid-upper-arm circumference, years of schooling, marital status, employment, religion, maternal capabilities, haemoglobin concentration, household Coping Strategy Index, proportion of household members practising open defecation, faeces observed in yard, household floor type, time to fetch drinking water, chicken ownership, livestock observed inside house, number of household occupants, wealth index quintile, infant low versus normal birthweight, infant sex, and preterm birth; the study factors data collector and calendar year of enrolment were also considered for inclusion.

Cumulative mortality at 18 months was 5·2% in the standard-of-care group, 4·2% in the IYCF group, 4·9% in the WASH group, and 4·9% in the IYCF plus WASH group, and did not differ significantly between groups. Treatment group effects on length-for-age Z scores, haemoglobin concentrations, stunting, anaemia, and diarrhoea prevalence were similar in all prespecified sensitivity analyses compared with those in the total analytic sample (data not shown). However, in the prespecified subgroup analysis by infant sex, boys had poorer linear growth than girls at 18 months, with lower mean length-for-age Z scores (–1·66 [95% CI −1·72 to −1·61] *vs* −1·35 [–1·40 to −1·30]) and a higher proportion of stunting (36·7% [34·4 to 39·0] *vs* 25·0% [23·1 to 27·2]). Child sex modified the effects of the IYCF intervention on length-for-age Z scores (p_interaction_=0·016) but not stunting (p_interaction_=0·420). The IYCF intervention was more efficacious in increasing mean length-for-age Z scores among boys (0·24 [95% CI 0·14 to 0·34]) than among girls (0·07 [95% CI −0·04 to 0·17]). Child sex did not modify the effect of the IYCF intervention on haemoglobin concentration or anaemia, or the effect of the WASH intervention on either primary outcome (data not shown).

Among 653 serious adverse events, none were judged to be related to the trial interventions (appendix). Three adverse events were judged to be related to trial interventions (one possibly related, one probably related, and one definitely related). In the IYCF group, a child with congenital abnormalities complained of abdominal discomfort after ingestion of the small-quantity lipid-based nutrient supplement (possibly related). In the IYCF plus WASH group, one child had diarrhoea after consumption of the small-quantity lipid-based nutrient supplement (probably related). In the WASH group, one child was fed WaterGuard by a sibling (definitely related). The child was reviewed at the clinic and treated with paracetamol for 3 days. All three cases resolved completely with no sequelae.

## Discussion

We tested the independent and combined effects of an infant feeding intervention and a household WASH intervention on attained child length and haemoglobin concentration at 18 months of age. Interventions were delivered with high fidelity of intervention and substantial contrast was achieved in WASH and IYCF hardware, commodities, and behaviours. Consistent with decades of complementary feeding research,^3^, ^22^ the IYCF interventions increased linear growth and haemoglobin concentrations, reduced stunting by 21%, reduced anaemia by 24%, and increased head circumference and ponderal growth compared with the non-IYCF interventions. Although we could not separate out the effects of complementary feeding education from those of the lipid-based nutrient supplement, our formative work showed that both components are important.^12^, ^13^ By contrast, we detected no benefit for the WASH intervention on any reported child health outcomes. Length-for-age Z scores, haemoglobin concentrations, proportions of stunted and anaemic children, and indicators of ponderal growth were similar in WASH and non-WASH groups. Head circumference at the 18-month visit was greater in the WASH than in the non-WASH groups in adjusted analyses, but this difference was driven by the IYCF plus WASH group, and was thus unlikely to be a true WASH effect.

The IYCF intervention did not decrease the 7-day prevalence of diarrhoea at either the 12-month or 18-month visits. We think that the increased diarrhoea prevalence in the IYCF group at 12 months was a chance occurrence. Although diarrhoea risk is moderately increased by oral iron when given as a medicinal supplement, this risk is nearly absent when iron is given as a food fortificant.^23^

The WASH intervention had no effect on diarrhoea prevalence at either the 12-month or 18-month visits. This finding is not consistent with those of 2015 Cochrane reviews on water chlorination^24^ and handwashing promotion,^25^ in which these interventions were estimated to reduce diarrhoea by about 25%. Most of the studies included in these reviews (and nearly all the studies which showed a significant effect on diarrhoea) had very high intervention doses—ie, daily-to-weekly contact between behaviour-change promoters and study participants—which was greater than the monthly contact delivered in SHINE. Although one water chlorination trial^26^ published since the 2015 Cochrane reviews showed a 36% reduction in diarrhoea with monthly behaviour change intervention contacts, at the midpoint between intervention visits staff visited to measure chlorine residuals—ie, participants received a visit about water chlorination every 2 weeks. Furthermore, several studies included in the Cochrane reviews showed no effect on diarrhoea, even with daily-to-weekly intervention doses. Finally, follow-up studies^27^, ^28^ suggest that the effect of these interventions on diarrhoea is not sustained once frequent intervention contacts end. Thus, adherence to handwashing and water-chlorination interventions (both highly dependent on sustained behaviour change) might not be sufficient to reduce diarrhoea when intervention dose is less frequent than monthly, even when behaviour-change messages are based on extensive formative research, delivered by highly trained workers, and accompanied by free provision of soap and chlorine, as in SHINE. Intervention dose is often not reported in WASH behaviour-change studies and, to our knowledge, has not been extracted in any systematic review of WASH studies,^24^, ^25^, ^29^, ^30^ suggesting that importance of very frequent sustained behaviour-change promotion for home-based water chlorination and handwashing promotion might not be widely recognised.

Several other aspects of the trial could be important. First, we intervened at the household rather than community level, because we reasoned that young children spend most of their time within their own household. Increased community sanitation coverage, even in sparsely populated areas, might be required to affect growth. Although open defecation was reduced in the WASH households from around 50% to less than 1%, we estimate that community-level open defecation in the WASH clusters was reduced from around 55% to around 40% (data not shown). Decreased open defecation^31^ and higher sanitation coverage^32^ at the community level have been associated with reduced stunting. A trial^33^ in Mali showed that communities randomised to a community-led sanitation-intervention had improved linear growth compared with control communities. Finally, although the SHINE WASH intervention considerably reduced geophagia and consumption of chicken faeces by maternal history, it did not prevent these behaviours (27% of WASH mothers still reported they had observed geophagia at the 12-month visit). Analyses of structured observation and in-depth interview data are underway, and will provide detailed information about how the play space was used and whether and by what magnitude this intervention interrupted faecal–oral microbial transmission due to child exploratory play.

SHINE is the third trial in which a WASH intervention alone or in combination with an IYCF intervention had no effect on linear growth.^34^, ^35^ Although these findings do not unequivocally prove that an integrated WASH–nutrition approach will never improve linear growth in any context, these trials included more than 18 000 children in three diverse settings where stunting is prevalent and environmental hygiene and infant diets are poor. We have three potential explanations for the lack of effects of WASH interventions on linear growth. First, our hypothesis could have been incorrect—perhaps reduction of faecal ingestion does not reduce environmental enteric dysfunction, or perhaps prevention of environmental enteric dysfunction does not improve linear growth. The cause and growth effects of environmental enteric dysfunction remain poorly understood. Laboratory analyses of biomarkers of environmental enteric dysfunction from the SHINE trial are underway.

Second, our hypothesis could have been correct, but the WASH interventions used were not effective enough to facilitate linear growth or, in two of the three trials, to reduce diarrhoea. Throughout history, linear growth and child health have improved after substantial socioeconomic development, as occurred after the industrial revolution in Europe and more recently in Latin America. These health benefits have been partly attributed to provision of piped water into homes, sewage systems, and flush toilets. For example, in Brazil, where stunting declined from 37% to 7% between 1996 and 2007, expansion of water supply and sanitation services, particularly for the poorest people (ie, in the lowest wealth quintile), was one of four factors credited for approximately two-thirds of the stunting decline.^36^ The absence of effects on linear growth and diarrhoea in SHINE suggests that the household-level interventions we implemented (point-of-use water chlorination, handwashing stations not connected to a water source, and improved pit latrines) might have little effect on child health, even on diarrhoea, unless the behaviour-change intervention is sustained daily or weekly, as implemented by the WASH Benefits Bangladesh trial and other efficacy trials.^34^ Moreover, the WASH Benefits Bangladesh trial suggested that even with this level of sustained intervention dose, these interventions might not be efficacious enough to improve growth. One important intervention untested in any of the three trials is provision of an on-plot, sustained, high-quality water supply—the aspirational goal of the Sustainable Development Goals (although most of the households in Bangladesh were less than 5 minutes' walk from an improved water source). In summary, to achieve and sustain diarrhoea prevention at scale and improve linear growth might require new, innovative interventions that are less dependent on behaviour change and more efficacious in reducing faecal exposure—a paradigm shift away from how rural WASH programmes are delivered.

Third, the trials did not address intergenerational prenatal factors. Already at 1 month of age, mean length-for-age Z score for infants in the SHINE trial was −0·85 (SD 1·25) and 16% were stunted (data not shown) despite high rates of early initiation and exclusive breastfeeding^16^ and installation of latrines and tippy taps during pregnancy. Preconception dietary supplementation of mothers (NCT01883193) and planned studies by our group to characterise the drivers of poor fetal growth during pregnancy could inform future preconception or prenatal interventions.

There is a large movement to scale up integrated WASH–nutrition interventions for stunting prevention.^37^ The SHINE trial provides high-level evidence that elementary WASH interventions delivered at the household level in rural areas of low-income and middle-income countries are unlikely to reduce stunting and might not reduce diarrhoea, and that implementation of these WASH interventions together with IYCF interventions will not reduce stunting more than implementation of IYCF alone. Our findings provide an urgent call for greater investment in the WASH sector to identify and deliver more efficacious interventions.
